# Supplementary material for: Distribution and Prevalence of Anaplasmataceae, Rickettsiaceae and Coxiellaceae in African Ticks: A Systematic Review and Meta-Analysis
Source: Microorganisms. 2023 Mar 9;11(3):714. doi: 10.3390/microorganisms11030714 (PMC10051480; doi:10.3390/microorganisms11030714)
Supplement: Supplementary file 1 [file microorganisms-11-00714-s001.zip › Table_S1.pdf]

Supplementary material S1. PRISMA checklist and additional checklist based on Migliavaca et al., 2020 guidelines

PRISMA 2020 STATEMENT CHECKLIST

Section and Topic	Ite m #	Checklist item	Location where item is reported in this study
TITLE			
Title	1	Identify the report as a systematic review.	Title
ABSTRACT			
Abstract	2	See the PRISMA 2020 for Abstracts checklist.	Abstract
INTRODUCTION			
Rationale	3	Describe the rationale for the review in the context of existing knowledge.	Introduction
Objectives	4	Provide an explicit statement of the objective(s) or question(s) the review addresses.	Introduction + Materials and methods
METHODS			
Eligibility criteria	5	Specify the inclusion and exclusion criteria for the review and how studies were grouped for the syntheses.	Materials and methods
Information sources	6	Specify all databases, registers, websites, organisations, reference lists and other sources searched or consulted to identify studies. Specify the date when each source was last searched or consulted.	Materials and methods
Search strategy	7	Present the full search strategies for all databases, registers and websites, including any filters and limits used.	Materials and methods
Selection process	8	Specify the methods used to decide whether a study met the inclusion criteria of the review, including how many reviewers screened each record and each report retrieved, whether they worked independently, and if applicable, details of automation tools used in the process.	Materials and methods
Data collection process	9	Specify the methods used to collect data from reports, including how many reviewers collected data from each report, whether they worked independently, any processes for obtaining or confirming data from study investigators, and if applicable, details of automation tools used in the process.	Materials and methods
Data items	10a	List and define all outcomes for which data were sought. Specify whether all results that were compatible with each outcome domain in each study were sought (e.g. for all measures, time points, analyses), and if not, the methods used to decide which results to collect.	Materials and methods
	10b	List and define all other variables for which data were sought (e.g. participant and intervention characteristics, funding sources). Describe any assumptions made about any missing or unclear information.	Materials and methods
Study risk of bias assessment	11	Specify the methods used to assess risk of bias in the included studies, including details of the tool(s) used, how many reviewers assessed each study and whether they worked independently, and if applicable, details of automation tools used in the process.	Materials and methods
Effect measures	12	Specify for each outcome the effect measure(s) (e.g. risk ratio, mean difference) used in the synthesis or presentation of results.	Materials and methods
Synthesis methods	13a	Describe the processes used to decide which studies were eligible for each synthesis (e.g. tabulating the study intervention characteristics and comparing against the planned groups for each synthesis (item #5)).	Materials and methods
	13b	Describe any methods required to prepare the data for presentation or synthesis, such as handling of missing summary statistics, or data conversions.	Materials and methods
	13c	Describe any methods used to tabulate or visually display results of individual studies and syntheses.	Materials and methods
	13d	Describe any methods used to synthesize results and provide a rationale for the choice(s). If meta-analysis was performed, describe the model(s), method(s) to identify the presence and extent of statistical heterogeneity, and software package(s) used.	Materials and methods
	13e	Describe any methods used to explore possible causes of heterogeneity among study results (e.g. subgroup analysis, meta-regression).	Materials and methods
	13f	Describe any sensitivity analyses conducted to assess robustness of the synthesized results.	Materials and methods
Reporting bias assessment	14	Describe any methods used to assess risk of bias due to missing results in a synthesis (arising from reporting biases).	Materials and methods
Certainty assessment	15	Describe any methods used to assess certainty (or confidence) in the body of evidence for an outcome.	Materials and methods
RESULTS			

Study selection	16a	Describe the results of the search and selection process, from the number of records identified in the search to the number of studies included in the review, ideally using a flow diagram.	Results
	16b	Cite studies that might appear to meet the inclusion criteria, but which were excluded, and explain why they were excluded.	Supporting information S2
Study characteristics	17	Cite each included study and present its characteristics.	Results
Risk of bias in studies	18	Present assessments of risk of bias for each included study.	Supporting information S5
Results of individual studies	19	For all outcomes, present, for each study: (a) summary statistics for each group (where appropriate) and (b) an effect estimate and its precision (e.g. confidence/credible interval), ideally using structured tables or plots.	Results
Results of syntheses	20a	For each synthesis, briefly summarise the characteristics and risk of bias among contributing studies.	Discussion
	20b	Present results of all statistical syntheses conducted. If meta-analysis was done, present for each the summary estimate and its precision (e.g. confidence/credible interval) and measures of statistical heterogeneity. If comparing groups, describe the direction of the effect.	Results + supporting information S3 and S4
	20c	Present results of all investigations of possible causes of heterogeneity among study results.	Results + supporting information S3 and S4
	20d	Present results of all sensitivity analyses conducted to assess the robustness of the synthesized results.	Results + supporting information S3 and S4
Reporting biases	21	Present assessments of risk of bias due to missing results (arising from reporting biases) for each synthesis assessed.	Results
Certainty of evidence	22	Present assessments of certainty (or confidence) in the body of evidence for each outcome assessed.	Discussion
DISCUSSION			
Discussion	23a	Provide a general interpretation of the results in the context of other evidence.	Discussion
	23b	Discuss any limitations of the evidence included in the review.	Discussion/ Conclusion
	23c	Discuss any limitations of the review processes used.	Discussion/ Conclusion
	23d	Discuss implications of the results for practice, policy, and future research.	Discussion/ Conclusion
OTHER INFORMATION			
Registration and protocol	24a	Provide registration information for the review, including register name and registration number, or state that the review was not registered.	PROSPERO
	24b	Indicate where the review protocol can be accessed, or state that a protocol was not prepared.	Appendix S1
	24c	Describe and explain any amendments to information provided at registration or in the protocol.	N/A
Support	25	Describe sources of financial or non-financial support for the review, and the role of the funders or sponsors in the review.	N/A
Competing interests	26	Declare any competing interests of review authors.	N/A
Availability of data, code and other materials	27	Report which of the following are publicly available and where they can be found: template data collection forms; data extracted from included studies; data used for all analyses; analytic code; any other materials used in the review.	Materials and methods

PRISMA 2020 FOR ABSTRACTS CHECKLIST

Section and Topic	Item #	Checklist item	Reported (Yes/No)
TITLE			
Title	1	Identify the report as a systematic review.	Yes
BACKGROUND			
Objectives	2	Provide an explicit statement of the main objective(s) or question(s) the review addresses.	Yes
METHODS			
Eligibility criteria	3	Specify the inclusion and exclusion criteria for the review.	Yes
Information sources	4	Specify the information sources (e.g. databases, registers) used to identify studies and the date when each was last searched.	Yes
Risk of bias	5	Specify the methods used to assess risk of bias in the included studies.	Yes
Synthesis of results	6	Specify the methods used to present and synthesise results.	Yes
RESULTS			
Included studies	7	Give the total number of included studies and participants and summarise relevant characteristics of studies.	Yes
Synthesis of results	8	Present results for main outcomes, preferably indicating the number of included studies and participants for each. If meta-analysis was done, report the summary estimate and confidence/credible interval. If comparing groups, indicate the direction of the effect (i.e. which group is favoured).	Yes
DISCUSSION			
Limitations of evidence	9	Provide a brief summary of the limitations of the evidence included in the review (e.g. study risk of bias, inconsistency and imprecision).	Yes
Interpretation	10	Provide a general interpretation of the results and important implications.	Yes
OTHER			
Funding	11	Specify the primary source of funding for the review.	No
Registration	12	Provide the register name and registration number.	No

CHECKLIST FOR SYSTEMATIC REVIEWS OF PREVALENCE (Migliavaca et al., 2020)	
**Characteristics**	**Description**
Number of naming authors	10
Protocol registry or publication	PROSPERO register
Use of a reporting guideline	PRISMA
	Systematic review of systematic reviews (Migliavaca et al., 2020)
External funding source	No
Number of databases searched	5
Databases searched	Scopus
	ScienceDirect
	OAIster
	Ovid
	PubMed
Search strategy presented	Full search strategy presented for all the databases
Number of studies included in the review	34
Quality assessment of individual studies	AXIS
Quality of the body of evidence	GRADE
Data synthesis	Qualitative and quantitive
CHECKLIST FOR META-ANALYSIS OF PREVALENCE	
Characteristics	Description
Method approach	Classic (inverse-variance method)
Model	Random-effects
Variance estimator	Hartung-Knapp Sidik-Jonkman
Transformation	Freeman-Tukey double arcsin
Heterogeneity assessment	Subgroup meta-analysis
	Higgins & Thompson’s I2 statistic
	Q-Test of Heterogeneity
	Meta-regression
	Prediction Interval
Publication bias	Visual inspection of the funnel plot
	Egger's regression test
Software	R studio
