# Supplementary material for: Distribution and Prevalence of Anaplasmataceae, Rickettsiaceae and Coxiellaceae in African Ticks: A Systematic Review and Meta-Analysis
Source: Microorganisms. 2023 Mar 9;11(3):714. doi: 10.3390/microorganisms11030714 (PMC10051480; doi:10.3390/microorganisms11030714)
Supplement: Supplementary file 1 [file microorganisms-11-00714-s001.zip › Table_S2.pdf]

Supplementary material S2. List of papers excluded during full-text examination and relevant exclusion criteria

Papers excluded during original search

1. Abdela et al., 2018 : ticks were tested for association but not screened for pathogens
2. Adamu et al., 2014 : screening only in dogs, not in ticks
3. Adjou et al., 2018 : no screening on ticks
4. Ahmadu et al., 2004 : no screening on ticks
5. Andrè et al., 2018 : study design not applicable (review)
6. Anifowose et al., 2020 : engorged ticks from cattles
7. Barker et al., 2018 : study design not applicable (review)
8. Beckley et al., 2016 : no ticks collected
9. Ben Said et al., 2018 : study design not applicable (review)
10. Ben Said et al., 2014 : no target population
11. Benaissa et al., 2017 : no target population
12. Berrelha et al., 2009 : data cannot be extracted (tick species not specified)
13. Bitam, 2012 : study design not applicable (review)
14. Bonilla et al., 2021 : study design not applicable (review)
15. Boucher et al., 2020 : no screening on ticks
16. Burgdorfer et al., 1973 : data cannot be extracted (tick species not specified) and the ticks are engorged
17. Burridge et al., 1975 : study design not applicable (review)
18. Buysse et al., 2020 : study design not applicable
19. Byamukama et al., 2021 : no tick sampling
20. Capponi et al., 1970 : full-text PDF not found
21. Chitanga et al., 2021 : ticks not identified to the species level + engorged ticks
22. Ciceroni et al., 1988 : study conducted outside Africa
23. Davies, 1993 : study design not applicable (review)

24. De Clercq et al., 2015 : topic not applicable
25. Ehounoud et al., 2017 : study design not applicable (review)
26. Elfving et al., 2010 : different study area (Europe)
27. Elliott et al., 2021 : no target population (chiggers and humans)
28. Fagir et al., 2014 : no pathogens screened
29. Harris et al., 2017 : study design not applicable (experimental study)
30. Harrison et al., 2011 : engorged ticks
31. Hornok et al., 2019 : cannot understand which samples are from Africa
32. Iweriebor et al., 2020 : tick species not specified
33. Immelman & Button 1973 : study design not applicable (review)
34. Jadin & Panier, 1953 : full-text PDF not found
35. Kamani et al., 2019 : study design not applicable (review)
36. Kamani et al., 2013 : data cannot be extracted (tick species not specified)
37. Kasozi et al., 2021 : no target population (ovines and caprines)
38. Kelly, 2001 : no information provided
39. Kelly & Mason, 1991 : study design not applicable (experimental study)
40. Kiel et al., 2006 : no study area (Florida)
41. Kimita et al., 2016 : topic not applicable (phylogenetic analysis)
42. Kisten et al., 2021 : topic not applicable
43. Knopf et al., 2002 : no screening on ticks
44. Kuttler, 1984 : study design not applicable (review)
45. Lange et al., 1992 : full-text PDF not found
46. Lorusso et al., 2016 : no target population
47. Magona et al., 2011 : no target population
48. Mahan et al., 1995 : study design not applicable (experimental study)
49. Mattioli et al., 1997 : no screening on ticks
50. Mbati et al., 2002 : no screening on ticks
51. Mdladla et al., 2016 : no target population (goat)
52. Mediannikov et al., 2014 : no data to extract

53. Mediannikov et al., 2014 : no data to extract + letter to editor
54. Mutai et al., 2013 : ticks not reported to the species level
55. Ngumi et al., 1997 : study design not applicable (experimental study)
56. Norval et al., 1984 : no target population
57. Norval et al., 1990 : no intervention (ticks not screened with laboratory methods)
58. Ouedraego et al., 2021 (b) : same samples of Ouedraego et al., 2021
59. Palomar et al., 2016 : information provided are insufficient
60. Parola et al., 2000 : information provided are insufficient
61. Pascucci et al., 2014 : study design not applicable (experimental study)
62. Peter et al., 1998 : study design not applicable (experimental study)
63. Peter et al., 2002 : study design not applicable (review)
64. Peter et al., 2000 : study design not applicable (experimental study)
65. Potgieter et al., 1983 : study design not applicable (experimental study)
66. Portillo et al., 2007 : engorged ticks + PDF not available
67. Pretorius et al., 2002 : study design not applicable (case report)
68. Prieto-Granada et al. : PDF and information not found
69. Reece et al. : PDF not found
70. Renè-Martellet et al., 2017 : study design and purpose not applicable
71. Ruszkowski et al. : study design not applicable (review)
72. Schoepf et al., 1984 : no target population (cattle)
73. Schreuder et al., 1977 : study design not applicable (experimental study)
74. Socolovschi et al., 2009 (a) : engorged ticks
75. Socolovschi et al., 2009 (b) : study design not applicable (experimental study)
76. Titcomb et al., 2017 : study design not applicable (experimental study)
77. Zemtsova et al., 2010 : study design not applicable (experimental study)
78. Van Vuuren & Penzhorn, 2015 : study design not applicable (review)
79. Sili et al., 2021 : no screening on ticks
80. Simpson et al., 2018 : no target population (human)

81. Tembo et al., 2018 : no screening on ticks

82. Van Heerden et al., 1995 : PDF and information not found

83. Smith et al., 1998 : study design not applicable (experimental study)

84. Waghela et al., 1991 : study design not applicable (experimental study)

85. Wang et al., 2009 : no target population

86. Younan et al., 2021 : no target population

87. Yunker et al., 1993 : study design not applicable (experimental study)

88. Zhang et al., 2016 : study area not applicable (China)

89. Znazen et al., 2011 : no target population

Papers excluded during search update

1. Broughton et al., 2021 : data cannot be extracted

2. Chigwada et al., 2022 : data not applicable (microbiome study)

3. Chitanga S et al., 2021 : already excluded in initial search

4. Iweriebor et al., 2022 : no outcome (screened only for piroplasmida)

5. Kisten et al., 2021 : already excluded in initial search

6. Mahlobo et al., 2021 : no outcome (screened only for piroplasmida)

7. Middlebrook et al., 2022 : study design not applicable (review)

8. Moustafa et al., 2022 : no study area (Japan)

9. Rjeibi et al., 2022 : engorged ticks
