# Supplementary material for: Distribution and Prevalence of Anaplasmataceae, Rickettsiaceae and Coxiellaceae in African Ticks: A Systematic Review and Meta-Analysis
Source: Microorganisms. 2023 Mar 9;11(3):714. doi: 10.3390/microorganisms11030714 (PMC10051480; doi:10.3390/microorganisms11030714)
Supplement: Supplementary file 1 [file microorganisms-11-00714-s001.zip › Table_S3_revised.pdf]

Pathogen_species	Sampling_country	Tick_species	References
Anaplasma bovis	Kenya	*Amblyomma gemma*	[65]
		Amblyomma variegatum	
		Hyalomma rufipes	
		Hyalomma truncatum	
		Rhipicephalus appendiculatus	[139]
		Rhipicephalus evertsi	[65]
		Rhipicephalus praetextatus	
		Rhipicephalus pulchellus	
	South Africa	*Rhipicephalus decoloratus*	[53]
		Rhipicephalus evertsi	
		Rhipicephalus evertsi	[71]
	Tunisia	*Hyalomma dromedarii*	[148]
Anaplasma centrale	Ethiopia	*Amblyomma lepidum*	[105]
		Amblyomma variegatum	
	Ivory Coast	*Amblyomma variegatum*	[55]
	South Africa	*Rhipicephalus gertrudae*	[53]
	Uganda	*Rhipicephalus pulchellus*	[54]
Anaplasma marginale	Algeria	*Rhipicephalus annulatus*	[89]
	Benin	*Amblyomma variegatum*	[183]
	Burkina Faso	*Rhipicephalus decoloratus*	[56]
	Egypt	*Hyalomma excavatum*	[142]
			[74]

		Rhipicephalus annulatus	[142]
			[141]
			[74]
		Rhipicephalus sanguineus	[145]
	Ethiopia	*Amblyomma cohaerens*	[116]
		Amblyomma lepidum	[105]
		Amblyomma variegatum	
	Guinea	*Rhipicephalus geigyi*	[121]
		Rhipicephalus microplus	
	Ivory Coast	*Rhipicephalus microplus*	[55]
	Kenya	*Rhipicephalus decoloratus*	[59]
	Madagascar	*Amblyomma variegatum*	[132]
		Rhipicephalus microplus	
	South Africa	*Rhipicephalus decoloratus*	[53]
			[115]
		Rhipicephalus evertsi	[53]
		Rhipicephalus gertrudae	
	United Republic of Tanzania	*Amblyomma gemma*	[114]
		Rhipicephalus appendiculatus	
		Rhipicephalus compositus	
		Rhipicephalus decoloratus	
		Rhipicephalus praetextatus	
		Rhipicephalus pulchellus	
	Zambia	*Argas walkerae*	[147]
Anaplasma ovis		*Rhipicephalus bursa*	[110]

	Algeria	*Rhipicephalus turanicus*	
	Ethiopia	*Rhipicephalus decoloratus*	[105]
		Rhipicephalus evertsi	
	Kenya	*Amblyomma gemma*	[65]
		Amblyomma variegatum	
		Rhipicephalus appendiculatus	
		Rhipicephalus appendiculatus	[139]
		Rhipicephalus decoloratus	[59]
		Rhipicephalus evertsi	
		Rhipicephalus evertsi	[139]
		Rhipicephalus pulchellus	[65]
	Mozambique	*Rhipicephalus microplus*	[103]
	South Africa	*Amblyomma hebraeum*	[71]
		Rhipicephalus evertsi	[53]
	Tunisia	*Rhipicephalus sanguineus*	[111]
		Rhipicephalus turanicus	
Anaplasma phagocytophilum	Algeria	*Argas persicus*	[89]
	Egypt	*Rhipicephalus sanguineus*	[145]
	Ethiopia	*Amblyomma cohaerens*	[116]
		Amblyomma lepidum	[105]
		Amblyomma variegatum	
	Kenya	*Rhipicephalus maculatus*	[127]
	South Africa	*Amblyomma hebraeum*	[124]
		Haemaphysalis elliptica	[125]

		Rhipicephalus decoloratus	[124]
		Rhipicephalus evertsi	
		Rhipicephalus sanguineus	[125]
	Tunisia	*Hyalomma detritum*	[104]
		Hyalomma marginatum	[123]
		Ixodes ricinus	[104]
	Zambia	*Ornithodoros moubata*	[147]
Anaplasma platys	Democratic Republic of the Congo	*Rhipicephalus sanguineus*	[133]
	Egypt	*Hyalomma excavatum*	[142]
		Rhipicephalus annulatus	
		Rhipicephalus annulatus	[141]
		Rhipicephalus sanguineus	[145]
	Ethiopia	*Rhipicephalus decoloratus*	[76]
	Guinea	*Rhipicephalus microplus*	[121]
	Kenya	*Rhipicephalus appendiculatus*	[59]
		Rhipicephalus camicasi	[122]
		Rhipicephalus decoloratus	[59]
		Rhipicephalus evertsi	[65]
		Rhipicephalus pravus	
		Rhipicephalus pulchellus	
	N/A	*Amblyomma variegatum*	[76]
		Rhipicephalus sanguineus	[122]
	South Africa	*Rhipicephalus evertsi*	[53]

	Tunisia	*Hyalomma dromedarii*	[134]
		Rhipicephalus sanguineus	[104]
Anaplasma spp.	Ivory Coast	*Amblyomma variegatum*	[55]
		Hyalomma truncatum	
		Rhipicephalus microplus	
	Tunisia	*Hyalomma dromedarii*	[148]
		Hyalomma excavatum	
	United Republic of Tanzania	*Amblyomma gemma*	[117]
Anaplasma spp. (Omatjenne)	Ethiopia	*Amblyomma lepidum*	[105]
		Amblyomma variegatum	
Candidatus Anaplasma ivorensis	Ivory Coast	*Amblyomma variegatum*	[55]
Candidatus Ehrlichia rustica	Egypt	*Rhipicephalus annulatus*	[141]
	Ivory Coast	*Amblyomma variegatum*	[55]
		Hyalomma truncatum	
		Rhipicephalus microplus	
Candidatus Ehrlichia urmitei		*Amblyomma variegatum*	
		Hyalomma truncatum	

		Rhipicephalus microplus	
Candidatus Rickettsia barbariae	Algeria	Hyalomma excavatum	[67]
		Rhipicephalus bursa	
	Swaziland	Rhipicephalus simus	[117]
Candidatus Rickettsia kastelanii	Morocco	Haemaphysalis punctata	[94]
		Haemaphysalis sulcata	
Coxiella burnetii	Algeria	Haemaphysalis erinacei	[143]
		Hyalomma excavatum	[67]
		Ixodes vespertilionis	[73]
		Rhipicephalus bursa	[67]
			[110]
		Rhipicephalus sanguineus	[143]
	Egypt	Argas persicus	[74]
		Hyalomma dromedarii	
		Hyalomma excavatum	
		Rhipicephalus sanguineus	
	Ethiopia	Amblyomma cohaerens	[62]
			[178]
			[182]
		Amblyomma gemma	[178]
		Amblyomma variegatum	[62]
			[178]

		[182]
	Hyalomma rufipes	[178]
	Rhipicephalus decoloratus	[62]
	Rhipicephalus decoloratus	[178]
	Rhipicephalus praetextatus	
	Rhipicephalus pulchellus	
Ghana	*Amblyomma variegatum*	[175]
	Hyalomma rufipes	
	Hyalomma truncatum	
	Rhipicephalus sanguineus	
	Rhipicephalus spp.	
Ivory Coast	*Amblyomma variegatum*	[55]
Kenya	*Amblyomma gemma*	[177]
	Amblyomma variegatum	[176]
	Haemaphysalis leachi	
	Rhipicephalus appendiculatus	
		[177]
		[181]
	Rhipicephalus decoloratus	[176]
	Rhipicephalus evertsi	[177]
		[181]
	Rhipicephalus pulchellus	[177]
		[181]
	Rhipicephalus sanguineus	[176]
N/A	*Rhipicephalus pulchellus*	[76]

	Nigeria	*Hyalomma dromedarii*	[168]
		Hyalomma truncatum	[168]
	Senegal	*Amblyomma variegatum*	[180]
		Hyalomma rufipes	[180]
		Hyalomma truncatum	[180]
		Ornithodoros sonrai	[180]
		Rhipicephalus annulatus	[180]
		Rhipicephalus decoloratus	[180]
		Rhipicephalus evertsi	[180]
		Rhipicephalus guilhoni	[180]
	South Africa	*Amblyomma hebraeum*	[71] [125]
		Haemaphysalis elliptica	[125]
		Rhipicephalus evertsi	[115]
		Rhipicephalus sanguineus	[125]
	Tunisia	*Hyalomma dromedarii*	[134]
		Hyalomma impeltatum	[134]
Coxiella spp.	Angola	*Amblyomma variegatum*	[146]
		Hyalomma truncatum	[146]
		Rhipicephalus decoloratus	[146]
		Rhipicephalus duttoni	[146]
		Rhipicephalus evertsi mimeticus	[146]
	Egypt	*Rhipicephalus sanguineus*	[76]
	Kenya	*Amblyomma cohaerens*	[76]
		Amblyomma gemma	[179]

		Amblyomma lepidum	
		Amblyomma personatum	[76]
		Amblyomma tholloni	
		Haemaphysalis spp.	
		Rhipicephalus appendiculatus	
		Rhipicephalus carnivoralis	
		Rhipicephalus compositus	
		Rhipicephalus maculatus	
		Rhipicephalus pravus	
		Rhipicephalus spp.	
	N/A	*Amblyomma gemma*	[76]
		Amblyomma variegatum	
		Hyalomma truncatum	
		Rhipicephalus evertsi	
		Rhipicephalus praetextatus	
		Rhipicephalus pulchellus	
	Namibia	*Argas transgariepinus*	[169]
	Nigeria	*Hyalomma dromedarii*	[168]
		Hyalomma truncatum	
Coxiella-like endosymbionts	Algeria	*Dermacentor marginatus*	[26]
		Haemaphysalis sulcata	
		Hyalomma detritum	
		Hyalomma excavatum	
		Hyalomma lusitanicum	
		Hyalomma marginatum	

	Egypt	*Rhipicephalus sanguineus*	[76]
	Kenya	*Amblyomma eburneum*	[127]
		Amblyomma personatum	[76]
		Amblyomma tholloni	
		Haemaphysalis spp.	
		Rhipicephalus appendiculatus	
		Rhipicephalus carnivoralis	
		Rhipicephalus compositus	
		Rhipicephalus maculatus	
		Rhipicephalus pravus	
		Rhipicephalus spp.	
	N/A	*Amblyomma gemma*	
		Amblyomma variegatum	
		Hyalomma truncatum	
		Rhipicephalus evertsi	
		Rhipicephalus praetextatus	
	Sao Tome and Principe	*Amblyomma astrion*	[157]
		Amblyomma variegatum	
Ehrlichia canis	Algeria	*Rhipicephalus sanguineus*	[82]
			[89]
	Burkina Faso		[137]

		[130]
Cameroon		
		[129]
Egypt		[145]
Ghana		[137]
Ivory Coast		[135]
	Rhipicephalus evertsi	
Kenya	*Rhipicephalus pravus*	[65]
	Rhipicephalus pulchellus	
Mali	*Rhipicephalus muhsamae*	[77]
Niger	*Hyalomma truncatum*	

	South Africa	*Rhipicephalus evertsi*	[71]
		Rhipicephalus sanguineus	[125]
Ehrlichia chaffeensis	Cameroon	*Rhipicephalus sanguineus*	[130]
			[129]
	Kenya	*Amblyomma spp.*	[127]
Ehrlichia chaffeensis-like	Egypt	*Hyalomma excavatum*	[142]
Ehrlichia ewingii	Cameroon	*Rhipicephalus sanguineus*	[130]
Ehrlichia minasensis	Egypt	*Hyalomma excavatum*	[142]
		Rhipicephalus annulatus	
	Swaziland	*Rhipicephalus muehlensi*	[117]
		Rhipicephalus simus	

	Algeria	*Haemaphysalis sulcata*	[89]
Ehrlichia muris	South Africa	*Rhipicephalus appendiculatus*	[140]
		Rhipicephalus evertsi	
		Rhipicephalus sanguineus	
Ehrlichia ovina		*Hyalomma rufipes*	[53]
		Rhipicephalus decoloratus	
		Rhipicephalus evertsi	
		Rhipicephalus gertrudae	
Ehrlichia ruminantium	Algeria	*Hyalomma aegyptium*	[144]
	Benin	*Amblyomma variegatum*	[183]

			[56]
		Rhipicephalus microplus	[50]
	Burkina Faso	*Amblyomma variegatum*	[106]
			[56]
		Rhipicephalus microplus	[50]
	Cameroon	*Amblyomma variegatum*	[51]
	Ethiopia	*Amblyomma cohaerens*	[116]
			[138]
		Amblyomma lepidum	[76]
			[105]

		Amblyomma variegatum	[116]
			[105]
		Rhipicephalus decoloratus	[105]
	Gambia	*Amblyomma variegatum*	[113]
	Ivory Coast	*Amblyomma variegatum*	[55]
		Rhipicephalus microplus	[50]
	Kenya	*Amblyomma falsomarmoreum*	[65]
		Amblyomma gemma	
		Amblyomma nuttalli	
		Amblyomma sparsum	

		Amblyomma variegatum	
		Hyalomma impeltatum	[76]
		Rhipicephalus evertsi	[65]
	N/A	*Amblyomma variegatum*	[76]
	South Africa	*Amblyomma hebraeum*	[109]
			[53]
			[112]
			[115]
			[63]
			[91]

		[131]
	Rhipicephalus decoloratus	[115]
Sudan	*Amblyomma lepidum*	[126]
	Amblyomma variegatum	
Swaziland	*Amblyomma hebraeum*	[117]
Uganda	*Amblyomma gemma*	[54]
	Amblyomma lepidum	
	Amblyomma variegatum	[54], [128]
	Hyalomma truncatum	[54]

	Zimbabwe	*Amblyomma hebraeum*	[120]
			[131]
Ehrlichia spp.	Angola	*Rhipicephalus decoloratus*	[146]
	Cameroon	*Rhipicephalus sanguineus*	[130]
			[129]
	Guinea	*Rhipicephalus annulatus*	[121]
		Rhipicephalus geigyi	
		Rhipicephalus microplus	
	Ivory Coast	*Amblyomma variegatum*	[55]
		Hyalomma truncatum	

		Rhipicephalus microplus	
	South Africa	*Amblyomma and Rhipicephalus spp.*	[107]
	United Republic of Tanzania	*Amblyomma gemma*	[117]
		Amblyomma lepidum	
		Amblyomma variegatum	
Ehrlichia spp. (EU191229.1)	South Africa	*Hyalomma rufipes*	[53]
		Rhipicephalus decoloratus	
		Rhipicephalus evertsi	
Ehrlichia/Anaplasma spp.	Algeria	*Argas persicus*	[89]
		Haemaphysalis erinacei	[143]
		Hyalomma aegyptium	[144]
		Hyalomma detritum	[89]

	Ixodes ricinus	
	Rhipicephalus annulatus	
	Rhipicephalus bursa	
	Rhipicephalus sanguineus	[143]
		[89]
Egypt	*Rhipicephalus annulatus*	[141]
Ethiopia	*Amblyomma lepidum*	[76]
	Rhipicephalus decoloratus	
		[105]
	Rhipicephalus evertsi	
	Ixodes aulacodi	[102]

	Ghana		
	Kenya	*Hyalomma impeltatum*	[76]
		Rhipicephalus pravus	[76]
	Morocco	*Ixodes ricinus*	[104]
	Mozambique	*Rhipicephalus microplus*	[103]
	N/A	*Amblyomma variegatum*	[76]
	South Africa	*Amblyomma hebraeum*	[53]
			[91]
		Haemaphysalis elliptica	[91]
		Hyalomma rufipes	[53]
		Rhipicephalus decoloratus	[53]

		Rhipicephalus evertsi	
		Rhipicephalus microplus	
		Rhipicephalus sanguineus	[91]
		Rhipicephalus warburtoni	[53]
	Tunisia	*Hyalomma detritum*	[104]
		Hyalomma dromedarii	[148]
		Hyalomma excavatum	
		Ixodes ricinus	[104]
		Rhipicephalus sanguineus	
		Haemaphysalis leachi	[93]

	Uganda	*Rhipicephalus praetextatus*	
	Zambia	*Argas walkerae*	[147]
		Ornithodoros faini	
		Ornithodoros moubata	
Neoehrlichia mikiurensis	Ethiopia	*Amblyomma cohaerens*	[138]
Neoehrlichia spp.	South Africa	*Rhipicephalus sanguineus*	[91]
Occidentia massiliensis	Senegal	*Ornithodoros sonrai*	[174]
Panola Mountain Ehrlichia (PME)	Zambia	*Amblyomma variegatum*	[119]
Rickettsia aeschlimanni	Algeria	*Hyalomma aegyptium*	[144]
			[153]
		Hyalomma detritum	[154]
		Hyalomma dromedarii	[70]
		Hyalomma excavatum	[67]
			[73]
		Hyalomma marginatum	[67]
			[154]
		Hyalomma scupense	[73]
	Angola	*Hyalomma truncatum*	[146]
	Burkina Faso	*Hyalomma rufipes*	[81]
		Hyalomma truncatum	

	Chad	*Hyalomma rufipes*	[75]
	Djibouti	*Hyalomma rufipes*	[75]
	Egypt	*Hyalomma dromedarii*	[74]
		Hyalomma impeltatum	
		Hyalomma rufipes	
	Ethiopia	*Hyalomma impeltatum*	[81]
		Hyalomma rufipes	
		Hyalomma truncatum	
	Ivory Coast	*Hyalomma marginatum*	[55]
		Hyalomma truncatum	
	Kenya	*Amblyomma variegatum*	[64]
		Hyalomma impeltatum	[76]
		Hyalomma marginatum	[64]
		Hyalomma rufipes	[76]
			[65]
		Hyalomma truncatum	[64]
			[65]
		Rhipicephalus pulchellus	[64]
			[65]
	Mali	*Hyalomma rufipes*	[77]
	Morocco	*Hyalomma marginatum*	[151]
			[94]
	Niger	*Hyalomma rufipes*	[77]
		Hyalomma impeltatum	[72]

	Nigeria	*Hyalomma rufipes*	
	Senegal	*Hyalomma impeltatum*	[78]
		Hyalomma rufipes	[163]
			[78]
		Hyalomma truncatum	[163]
		Rhipicephalus evertsi	
		Rhipicephalus evertsi	[78]
	South Africa	*Hyalomma rufipes*	[71]
		Rhipicephalus appendiculatus	
		Rhipicephalus evertsi	
	Sudan	*Hyalomma rufipes*	[80]
	Tunisia	*Hyalomma dromedarii*	[69]
		Hyalomma dromedarii	[79]
		Hyalomma impeltatum	
Rickettsia africae	Algeria	*Hyalomma aegyptium*	[144]
		Hyalomma aegyptium	[89]
		Hyalomma detritum	
		Hyalomma dromedarii	[90]
	Angola	*Amblyomma variegatum*	[150]
			[146]
		Rhipicephalus decoloratus	[146]
	Burkina Faso	*Amblyomma variegatum*	[81]
	Burundi	*Amblyomma variegatum*	[77]

	Central African Republic	*Amblyomma variegatum*	[60]
		Rhipicephalus compositus	
		Rhipicephalus muhsamae	
	Comoros	*Amblyomma variegatum*	[66]
		Rhipicephalus appendiculatus	
		Rhipicephalus microplus	
	Democratic Republic of the Congo	*Amblyomma compressum*	[163]
	Djibouti	*Amblyomma lepidum*	[75]
			[96]
		Amblyomma variegatum	[75]
	Ethiopia	*Amblyomma cohaerens*	[62]
			[138]
		Amblyomma gemma	[81]
		Amblyomma lepidum	[62]
		Amblyomma variegatum	[62]
			[138]
		Rhipicephalus decoloratus	[105]
		Rhipicephalus evertsi	
	Guinea	*Amblyomma variegatum*	[163]
		Haemaphysalis paraleachi	
		Hyalomma rufipes	
		Rhipicephalus annulatus	
		Rhipicephalus decoloratus	
		Amblyomma variegatum	[55]

		Hyalomma impressum	
	Ivory Coast	*Hyalomma marginatum*	
		Hyalomma truncatum	
		Rhipicephalus microplus	
	Kenya	*Amblyomma eburneum*	[164]
			[127]
		Amblyomma gemma	[59]
			[64]
			[65]
			[139]
		Amblyomma lepidum	[64]
		Amblyomma variegatum	[59]
			[160]
			[92]
			[65]
			[139]
		Hyalomma impeltatum	[76]
		Hyalomma truncatum	[64]
		Rhipicephalus appendiculatus	[59]
			[160]
		Rhipicephalus decoloratus	[59]
		Rhipicephalus evertsi	[65]
		Rhipicephalus pulchellus	
	Liberia	*Amblyomma compressum*	[163]
		Amblyomma variegatum	

		Rhipicephalus geigyi	
	Mali	*Amblyomma variegatum*	[77]
	Morocco	*Hyalomma aegyptium*	[167]
	Mozambique	*Amblyomma hebraeum*	[161]
		Amblyomma variegatum	[161]
			[103]
	N/A	*Amblyomma gemma*	[76]
		Amblyomma variegatum	
	Niger	*Amblyomma variegatum*	[77]
	Nigeria	*Amblyomma variegatum*	[159]
	Senegal	*Amblyomma variegatum*	[78]
		Hyalomma rufipes	
		Rhipicephalus annulatus	[163]
		Rhipicephalus evertsi	
	South Africa	*Amblyomma hebraeum*	[61]
			[71]
			[63]
			[91]
		Haemaphysalis elliptica	[91]
		Rhipicephalus appendiculatus	[71]
		Rhipicephalus evertsi	
	Sudan	*Amblyomma lepidum*	[77]
		Hyalomma dromedarii	[80]

	Swaziland	*Amblyomma hebraeum*	[117]
	Tunisia	*Hyalomma dromedarii*	[79]
		Hyalomma impeltatum	
	Uganda	*Amblyomma variegatum*	[159]
Rickettsia africae São Tomé	Sao Tome and Principe		[157]
Rickettsia akari	South Africa	*Rhipicephalus sanguineus*	[140]
		Rhipicephalus spp.	
Rickettsia conorii	Algeria	*Rhipicephalus sanguineus*	[82]
			[154]
	Central African Republic	*Rhipicephalus muhsamae*	[60]
	Kenya	*Rhipicephalus appendiculatus*	[64]
	Morocco	*Rhipicephalus sanguineus*	[83]
	Nigeria		[84]
	Senegal	*Rhipicephalus evertsi*	[163]
	Swaziland	*Haemaphysalis elliptica*	[117]
	Tunisia	*Rhipicephalus sanguineus*	[86]
			[87]
	Uganda	*Haemaphysalis leachi*	[93]
		Haemaphysalis punctaleachi	[96]
Rickettsia conorii ssp. caspia	Zambia	*Rhipicephalus sanguineus*	[155]
Rickettsia helvetica	Algeria	*Ixodes ricinus*	[90]
	Morocco		[94]

	Tunisia	*Hyalomma impeltatum*	[79]
		Ixodes ricinus	[95]
Rickettsia hoogstraalii	Namibia	*Argas transgariepinus*	[169]
	Zambia	*Argas walkerae*	[147]
Rickettsia israelensis	Tunisia	*Rhipicephalus sanguineus*	[87]
Rickettsia japonica	Sao Tome and Principe	*Amblyomma variegatum*	[157]
Rickettsia lusitaniae	Zambia	*Ornithodoros faini*	[147]
		Ornithodoros porcinus	[171]
Rickettsia massiliae	Algeria	*Rhipicephalus bursa*	[73]
		Rhipicephalus sanguineus	[143]
			[82]
			[154]
			[85]
			[73]
	Burkina Faso		[137]
	Central African Republic	*Haemaphysalis leachi*	[60]
		Rhipicephalus lunulatus	
		Rhipicephalus muhsamae	
		Rhipicephalus senegalensis	
		Rhipicephalus sulcatus	
	Guinea	*Haemaphysalis paraleachi*	[163]
		Rhipicephalus senegalensis	
	Ivory Coast		[55]
	Mali	*Rhipicephalus muhsamae*	[77]

	Morocco	*Rhipicephalus sanguineus*	[83]
			[94]
	N/A	*Rhipicephalus praetextatus*	[76]
	Nigeria	*Rhipicephalus turanicus*	[172]
	Senegal	*Rhipicephalus guilhoni*	[163]
	South Africa	*Amblyomma sylvaticum*	[61]
		Rhipicephalus simus	
	Swaziland	*Haemaphysalis elliptica*	[117]
		Rhipicephalus simus	
	Tunisia	*Rhipicephalus sanguineus*	[87]
	Uganda	*Haemaphysalis leachi*	[93]
Rickettsia monacensis	Algeria	*Ixodes ricinus*	[152]
			[89]
			[98]
			[90]
	Morocco		[83]
			[94]
	Tunisia	*Hyalomma impeltatum*	[79]
		Ixodes ricinus	[95]
Rickettsia mongolotimonae	Algeria	*Hyalomma aegyptium*	[167]
	Kenya	*Hyalomma unidentified*	[64]
	Senegal	*Hyalomma truncatum*	[163]
Rickettsia montanensis	Kenya	*Rhipicephalus appendiculatus*	[64]
Rickettsia parkeri		*Hyalomma truncatum*	

		Rhipicephalus pulchellus	
Rickettsia raoultii	Liberia	*Ixodes muniensis*	[163]
	Morocco	*Dermacentor marginatus*	[94]
Rickettsia rhipicephali	Central African Republic	*Rhipicephalus compositus*	[60]
		Rhipicephalus lunulatus	
	Kenya	*Amblyomma cohaerens*	[76]
		Rhipicephalus evertsi	[65]
		Rhipicephalus pulchellus	
Rickettsia slovaca	Algeria	*Dermacentor marginatus*	[90]
			[73]
		Haemaphysalis punctata	
	Morocco	*Dermacentor marginatus*	[94]
Rickettsia sp. Ae-8	South Africa	*Amblyomma exornatum*	[173]
Rickettsia spp.	Algeria	*Carios capensis*	[13]
		Dermacentor marginatus	[90]
			[73]
		Haemaphysalis erinacei	[143]
			[85]
		Haemaphysalis punctata	[73]
		Haemaphysalis sulcata	[89]
		Hyalomma aegyptium	[144]
			[167]
		Hyalomma dromedarii	[70]
			[90]

	Hyalomma excavatum	[73]
	Hyalomma impeltatum	[70]
	Hyalomma rufipes	
	Hyalomma scupense	[73]
	Ixodes ricinus	[90]
	Ornithodoros erraticus	[13]
	Ornithodoros rupestris	
	Rhipicephalus bursa	[73]
	Rhipicephalus sanguineus	[143]
		[89]
		[85]
		[73]
Benin	*Amblyomma variegatum*	[108]
Cameroon		[170]
	Hyalomma rufipes	
	Hyalomma truncatum	
	Rhipicephalus lunulatus	
Central African Republic	*Amblyomma tholloni*	[162]
	Amblyomma variegatum	[60]
	Haemaphysalis leachi	
	Rhipicephalus compositus	
	Rhipicephalus lunulatus	
	Rhipicephalus muhsamae	
	Rhipicephalus senegalensis	
	Rhipicephalus sulcatus	

Chad	*Hyalomma rufipes*	[75]
Democratic Republic of the Congo	*Amblyomma compressum*	[163]
Djibouti	*Amblyomma lepidum*	[75]
		[96]
	Amblyomma variegatum	[75]
	Hyalomma rufipes	
Egypt	*Hyalomma dromedarii*	[149]
	Hyalomma impeltatum	
	Hyalomma marginatum	
Ethiopia	*Amblyomma cohaerens*	[62]
	Amblyomma lepidum	
	Amblyomma variegatum	
	Rhipicephalus decoloratus	
		[105]
	Rhipicephalus evertsi	
Gabon	*Amblyomma tholloni*	[162]
Ghana	*Amblyomma variegatum*	[175]
	Hyalomma rufipes	
	Hyalomma truncatum	
	Rhipicephalus evertsi	
	Rhipicephalus sanguineus	
	Rhipicephalus spp.	
Guinea	*Amblyomma variegatum*	[163]
	Haemaphysalis paraleachi	

		Hyalomma rufipes	
		Rhipicephalus annulatus	
		Rhipicephalus decoloratus	
		Rhipicephalus senegalensis	
	Ivory Coast	*Amblyomma variegatum*	[55]
		Hyalomma impressum	
		Hyalomma marginatum	
		Hyalomma truncatum	
		Rhipicephalus microplus	
		Rhipicephalus senegalensis	
	Kenya	*Amblyomma cohaerens*	[76]
		Amblyomma variegatum	[160]
			[92]
		Haemaphysalis leachi	
		Hyalomma impeltatum	[76]
		Hyalomma rufipes	
		Rhipicephalus appendiculatus	[160]
			[92]
		Rhipicephalus decoloratus	
		Rhipicephalus maculatus	[164]
		Rhipicephalus spp.	[92]
	Liberia	*Amblyomma compressum*	[163]
		Amblyomma variegatum	
		Ixodes muniensis	
		Rhipicephalus geigyi	

	Madagascar	*Amblyomma variegatum*	[158]
	Morocco	*Dermacentor marginatus*	[94]
		Haemaphysalis punctata	
		Haemaphysalis sulcata	
		Hyalomma aegyptium	[167]
		Hyalomma marginatum	[94]
		Ixodes ricinus	
		Rhipicephalus sanguineus	
	Mozambique	*Amblyomma hebraeum*	[161]
		Amblyomma variegatum	
			[103]
	N/A	*Amblyomma gemma*	[76]
		Amblyomma variegatum	
		Rhipicephalus praetextatus	
	Namibia	*Argas transgariepinus*	[169]
	Nigeria	*Amblyomma variegatum*	[159]
		Hyalomma dromedarii	[72]
			[168]
		Hyalomma impeltatum	[72]
			[168]
		Hyalomma rufipes	[72]
			[168]
		Hyalomma truncatum	
		Rhipicephalus sanguineus	[84]

Sao Tome and Principe	*Amblyomma astrion*	[157]
	Amblyomma variegatum	
Senegal	*Amblyomma variegatum*	[78]
	Hyalomma impeltatum	
	Hyalomma rufipes	[163]
		[78]
	Hyalomma truncatum	[163]
	Rhipicephalus annulatus	
	Rhipicephalus evertsi	
		[78]
	Rhipicephalus guilhoni	[163]
South Africa	*Amblyomma hebraeum*	[115]
		[61]
		[91]
	Amblyomma sylvaticum	[61]
	Haemaphysalis elliptica	[91]
	Rhipicephalus decoloratus	[115]
	Rhipicephalus evertsi	
	Rhipicephalus sanguineus	[91]
		[125]
	Rhipicephalus simus	[61]
		[71]
		[91]
Sudan	*Amblyomma variegatum*	[166]

	Tunisia	*Hyalomma dromedarii*	[69]
		Hyalomma impeltatum	[79]
		Ixodes ricinus	[95]
		Rhipicephalus sanguineus	[86]
			[87]
	Uganda	*Amblyomma variegatum*	[159]
			[165]
		Haemaphysalis leachi	[93]
		Haemaphysalis punctaleachi	[96]
		Rhipicephalus praetextatus	[93]
	United Republic of Tanzania	*Amblyomma gemma*	
		Amblyomma lepidum	[117]
		Amblyomma marmoreum	
		Amblyomma variegatum	
		Ornithodoros moubata	[156]
		Rhipicephalus appendiculatus	
		Rhipicephalus evertsi	
		Rhipicephalus muhsamae	[117]
		Rhipicephalus pravus	
		Rhipicephalus pulchellus	
		Rhipicephalus simus	
	Zambia	*Argas walkerae*	[147]
		Ornithodoros faini	
		Amblyomma hebraeum	[88]

	Zimbabwe	*Amblyomma rhinocerotis*	
		Amblyomma sparsum	
		Amblyomma variegatum	
		Haemaphysalis leachi	
		Hyalomma marginatum	
		Hyalomma truncatum	
		Rhipicephalus simus	
Rickettsia spp. (Davousti)	Gabon	*Amblyomma tholloni*	[162]
Rickettsia spp. (Uilenbergi)	Central African Republic		
Rickettsiella spp.	Namibia	*Argas transgariepinus*	[169]
SFG Rickettsia spp.	Algeria	*Ixodes ricinus*	[89]
		Rhipicephalus sanguineus	[82]
	Benin	*Amblyomma variegatum*	[108]
	Tunisia	*Rhipicephalus sanguineus*	[86]
