# Supplementary material for: Distribution and Prevalence of Anaplasmataceae, Rickettsiaceae and Coxiellaceae in African Ticks: A Systematic Review and Meta-Analysis
Source: Microorganisms. 2023 Mar 9;11(3):714. doi: 10.3390/microorganisms11030714 (PMC10051480; doi:10.3390/microorganisms11030714)
Supplement: Supplementary file 1 [file microorganisms-11-00714-s001.zip › Table_S4.pdf]

Pub_authors	Q1	Q2	Q3	Q4	Q5	Q6	Q8	Q9	Q10	Q11	Q12	Q15	Q16	Q17	Q18	Q19	Q20	TOT	Risk bias
Abdel-Shafy et al., 2012	1	0	0	1	0	0	1	1	0	1	1	1	1	1	0	1	0	0.5882353	Moderate
Abdelkadir et al., 2019	1	1	0	1	1	0	1	1	0	1	0	1	1	1	0	1	0	0.6470588	Moderate
Adakal et al., 2010	1	1	0	1	1	0	1	1	1	1	1	1	1	1	0	1	0	0.7647059	Low
Adelabu et al., 2020	1	1	0	1	1	0	1	1	0	1	0	1	1	1	0	1	1	0.7058824	Low
Adenyo et al., 2020	1	0	0	0	0	0	1	1	0	1	1	1	1	1	1	1	0	0.5882353	Moderate
Adjou Moumouni et al., 2018	1	1	0	1	1	0	1	1	1	1	1	1	1	1	1	1	1	0.8823529	Low
Adjou Moumouni et al., 2016	1	1	0	1	1	0	1	1	1	1	1	1	1	1	0	0	1	0.7647059	Low
Allsopp et al., 1999	1	1	0	1	0	1	1	1	0	1	1	1	1	1	1	0	0	0.7058824	Low
Aouadi et al., 2017	1	1	0	1	1	1	1	1	0	1	1	1	1	1	0	1	0	0.7647059	Low
Barradas et al., 2021	1	1	0	1	1	0	1	1	0	1	1	1	1	1	0	1	0	0.7058824	Low
Beati et al., 1995	1	0	0	1	0	0	1	1	0	1	1	1	1	1	1	1	0	0.6470588	Moderate
Beati et al., 1997	0	0	0	1	0	0	1	1	0	1	1	1	1	1	0	0	0	0.4705882	High
Belkhahia et al., 2019	1	1	0	1	0	0	1	1	0	1	0	1	1	1	0	1	0	0.5882353	Moderate
Benredjem et al., 2014	0	0	0	1	0	0	0	1	0	0	0	1	0	0	0	0	0	0.1764706	High
Berggoetz et al., 2014 (b)	1	1	0	1	1	0	1	1	1	1	0	1	0	1	0	1	0	0.6470588	Moderate
Bessas et al., 2016	1	0	0	1	0	0	1	1	0	1	1	1	1	1	0	1	1	0.6470588	Moderate
Biguezoton et al., 2016	1	0	0	1	0	0	1	1	0	1	0	1	1	1	0	1	1	0.5882353	Moderate
Bitam et al., 2009	0	0	0	0	0	0	0	1	0	0	1	1	1	1	0	0	0	0.2941176	High
Bitam et al., 2006	1	0	0	0	0	0	1	1	0	0	0	0	1	1	0	0	0	0.2941176	High
Boucheikhchoukh et al., 2018	0	0	0	1	0	0	0	1	0	1	0	0	1	1	1	1	1	0.4705882	High
Boudebouch et al., 2009	0	0	0	0	0	0	0	1	0	1	1	1	1	1	0	1	0	0.4117647	High
Bryson et al., 2002	1	0	0	1	0	0	0	1	0	1	1	1	1	1	0	1	0	0.5294118	Moderate
Byaruhanga et al., 2021	1	0	0	1	0	1	1	1	0	1	0	1	1	1	1	1	1	0.7058824	Low
Chitimia-Dobler et al., 2017	0	0	0	0	0	0	0	1	0	0	0	1	0	1	0	0	0	0.1764706	High
Chiuya et al., 2021	0	1	0	1	1	1	1	1	0	1	1	1	1	1	0	1	1	0.7647059	Low
Cutler et al., 2006	0	1	0	1	1	0	1	1	0	0	0	1	0	1	0	0	0	0.4117647	High
Demoncheaux et al., 2012	1	0	0	1	0	0	1	1	0	1	1	1	1	1	0	0	0	0.5294118	Moderate
Dib et al., 2009	1	0	0	0	0	0	1	1	0	1	1	1	1	1	0	0	0	0.4705882	High
Djerbouh et al., 2012	1	1	0	1	1	0	1	1	0	0	1	1	1	1	0	0	0	0.5882353	Moderate
Dupont et al., 1994	1	1	0	1	1	0	1	1	0	1	0	1	0	1	0	0	0	0.5294118	Moderate
Ehounoud et al., 2016	1	1	0	1	1	0	1	1	0	1	1	1	1	1	1	1	1	0.8235294	Low
Esemu et al., 2013	0	1	0	1	1	0	0	1	1	1	1	1	1	1	1	0	0	0.6470588	Moderate
Faburay et al., 2007	1	1	0	1	1	1	1	1	0	1	0	1	0	1	0	0	0	0.5882353	Moderate
Fyumagwa et al., 2009	1	1	0	1	1	1	1	1	0	1	0	1	1	1	0	0	0	0.6470588	Moderate
Guo et al., 2019	1	0	0	1	0	0	1	1	0	1	0	1	1	1	0	0	1	0.5294118	Moderate
Halajian et al., 2016	1	0	0	1	0	0	1	1	0	1	0	1	0	1	1	0	0	0.4705882	High
Halajian et al., 2018	1	0	0	1	0	0	1	1	0	0	0	1	1	1	0	1	0	0.4705882	High
Hornok et al., 2014	1	1	0	1	0	0	1	1	1	1	1	1	1	1	0	0	1	0.7058824	Low
Hornok et al., 2016	1	1	0	1	0	0	1	1	1	1	0	1	0	0	0	0	0	0.4705882	High
Hsi et al., 2020	1	0	0	1	0	1	1	1	0	1	1	1	1	1	0	1	1	0.7058824	Low

Pub_authors	Q1	Q2	Q3	Q4	Q5	Q6	Q8	Q9	Q10	Q11	Q12	Q15	Q16	Q17	Q18	Q19	Q20	TOT	Risk bias
Jonjejan et al., 2020	1	1	0	1	0	1	1	1	1	1	1	1	1	1	1	1	1	0.8823529	Low
Kamani et al., 2015	1	1	0	1	0	1	1	1	0	1	0	1	1	0	0	0	0	0.5294118	Moderate
Kamani et al., 2018	1	0	0	1	0	0	1	1	0	0	1	1	1	1	0	1	1	0.5882353	Moderate
Keller et al., 2016	0	0	0	1	0	1	0	1	0	1	1	1	1	1	1	1	1	0.6470588	Moderate
Kernif et al., 2012 (a)	1	1	0	1	0	0	1	1	0	1	1	1	1	1	0	1	0	0.6470588	Moderate
Kernif et al., 2012 (b)	1	1	0	0	0	0	1	1	0	1	1	1	1	1	0	0	0	0.5294118	Moderate
Khaldi et al., 2012	1	1	0	1	0	0	1	1	0	1	1	1	1	1	0	1	1	0.7058824	Low
Khrouf et al., 2014	1	1	0	1	1	0	1	1	1	1	1	1	1	1	0	1	0	0.7647059	Low
Kim et al., 2018	0	0	0	1	0	0	0	1	1	1	1	1	0	1	0	1	0	0.4705882	High
Knobel et al., 2013	1	1	0	1	1	1	1	1	0	1	1	1	1	1	0	0	1	0.7647059	Low
Koka et al., 2018	0	0	0	0	0	0	0	0	0	1	0	0	1	1	1	1	1	0.3529412	High
Koka et al., 2017	1	1	0	1	1	0	1	1	0	1	0	1	0	0	1	0	0	0.5294118	Moderate
Kolo et al., 2016	1	1	0	1	0	0	1	1	0	1	1	1	1	1	0	1	1	0.7058824	Low
Kumsa et al., 2015	1	1	0	1	1	0	1	1	1	1	1	1	1	1	1	0	1	0.8235294	Low
Lafri et al., 2015	0	0	0	1	0	0	0	1	0	1	0	1	0	1	0	1	0	0.3529412	High
Langguth et al., 2017	0	0	0	1	0	0	0	1	0	1	0	1	1	1	0	1	0	0.4117647	High
Ledger et al., 2021	1	1	0	1	1	0	1	1	1	0	0	1	1	1	1	1	0	0.7058824	Low
Leulmi et al., 2016	1	1	0	1	0	0	1	1	0	1	1	1	1	1	1	1	1	0.7647059	Low
Loftis et al., 2016	0	0	0	1	1	0	0	1	0	1	1	1	1	1	1	0	0	0.5294118	Moderate
Loftis et al., 2006	1	1	0	0	1	0	1	1	0	0	0	1	1	1	1	0	0	0.5294118	Moderate
Lorusso et al., 2013	1	0	0	1	1	0	0	1	0	1	0	1	1	1	0	0	1	0.5294118	Moderate
Macaluso et al., 2003	1	1	0	1	0	0	1	1	0	1	1	1	1	1	0	0	0	0.5882353	Moderate
Machado-Ferreira et al., 2016	0	0	0	1	0	0	0	0	0	1	0	1	1	1	0	0	0	0.2941176	High
Magaia et al., 2020	0	0	0	1	1	0	0	1	0	1	0	1	1	1	1	1	0	0.5294118	Moderate
Mahan et al., 1998	0	0	0	0	0	0	0	0	0	0	0	1	1	1	1	0	0	0.2352941	High
Maina et al., 2014	0	0	0	1	1	1	0	1	0	1	1	1	1	1	0	1	1	0.6470588	Moderate
Makenov et al., 2021	0	0	0	1	0	0	0	1	0	0	0	1	1	0	0	1	1	0.3529412	High
Matei et al., 2016	1	1	0	0	0	0	0	1	1	1	0	1	0	1	0	1	1	0.5294118	Moderate
Matsimbe et al., 2017	1	1	0	0	0	0	1	1	0	1	1	1	0	1	0	0	0	0.4705882	High
Matsumoto et al., 2007	0	0	0	1	0	0	0	1	0	1	1	1	1	1	0	0	0	0.4117647	High
Mediannikov et al., 2013	1	1	0	0	1	0	1	1	1	1	1	1	1	1	0	0	1	0.7058824	Low
Mediannikov et al., 2012 (a)	0	0	0	1	0	0	0	1	0	1	1	1	1	1	0	0	0	0.4117647	High
Mediannikov et al., 2012 (b)	0	0	0	1	1	0	1	1	0	1	1	1	1	1	0	0	0	0.5294118	Moderate
Mediannikov et al., 2010	1	1	0	1	1	0	1	1	1	1	1	1	1	1	0	1	1	0.8235294	Low
M'ghirbi et al., 2012	1	1	0	1	0	1	1	1	1	1	0	1	1	1	0	1	1	0.7647059	Low
Mtshali et al., 2016	1	1	0	1	0	0	1	1	0	0	0	1	0	1	0	0	1	0.4705882	High
Mtshali et al., 2017	1	1	0	1	0	0	0	1	1	0	0	1	0	1	0	1	0	0.4705882	High
Mura et al., 2008	1	1	0	1	0	0	1	1	0	1	1	1	1	1	0	1	1	0.7058824	Low
Muramatsu et al., 2005	1	1	0	1	0	0	1	1	0	0	1	1	1	1	0	0	0	0.5294118	Moderate
Mwamuye et al., 2016	1	1	0	1	0	0	1	1	0	0	0	1	0	1	0	0	0	0.4117647	High

Pub_authors	Q1	Q2	Q3	Q4	Q5	Q6	Q8	Q9	Q10	Q11	Q12	Q15	Q16	Q17	Q18	Q19	Q20	TOT	Risk bias
Mwamuye et al., 2017	1	1	0	1	0	0	1	1	0	1	0	1	0	1	1	0	0	0.5294118	Moderate
Nakao et al., 2013	1	1	0	1	1	1	1	1	0	0	1	1	1	1	0	0	0	0.6470588	Moderate
Nakao et al., 2015	1	1	0	1	1	0	1	1	0	1	1	1	1	1	0	1	0	0.7058824	Low
Nakao et al., 2010	1	1	0	1	1	1	1	1	1	1	1	1	1	1	1	0	0	0.8235294	Low
Ndeereh et al., 2017 (a)	1	1	0	1	1	0	1	1	1	1	1	1	0	1	0	1	1	0.7647059	Low
Ndip et al., 2010	1	1	0	1	0	0	1	1	0	0	1	1	1	1	0	0	0	0.5294118	Moderate
Ndip et al., 2007	1	1	0	1	0	0	1	1	0	1	0	1	1	1	0	0	0	0.5294118	Moderate
Norte et al., 2021	1	1	0	1	1	0	1	1	1	1	1	1	1	1	0	1	1	0.8235294	Low
Olivieri et al., 2021	1	1	0	1	1	0	1	1	1	1	1	0	1	1	0	1	1	0.7647059	Low
Omondi et al., 2017	1	1	0	1	0	0	1	1	0	1	1	1	1	1	0	1	1	0.7058824	Low
Onyiche et al., 2020	1	1	0	1	0	0	1	1	0	0	0	1	0	1	0	1	1	0.5294118	Moderate
Ouedraogo et al., 2021	1	1	0	1	1	1	1	1	1	1	0	1	0	1	1	1	1	0.8235294	Low
Parola et al., 2001	0	0	0	0	0	0	0	1	0	0	0	1	0	0	0	0	0	0.1176471	High
Peter et al., 1999 (a)	1	1	1	1	0	1	1	1	1	1	1	1	1	1	1	0	0	0.8235294	Low
Peter et al., 1999 (b)	1	1	0	1	0	0	0	1	1	1	1	1	1	1	0	0	0	0.5882353	Moderate
Pothmann et al., 2016	1	1	0	1	1	1	1	1	1	1	1	1	1	1	0	0	0	0.7647059	Low
Proboste et al., 2015	1	1	0	1	0	0	1	1	1	1	1	1	0	1	1	1	0	0.7058824	Low
Rahal et al., 2020	1	1	0	1	1	1	1	1	1	1	0	1	0	1	0	1	1	0.7647059	Low
Reeves et al., 2020	0	0	0	1	0	0	0	1	0	1	1	1	1	1	1	0	0	0.4705882	High
Sambou et al., 2014	1	1	0	1	1	0	1	1	0	1	1	1	1	1	0	1	0	0.7058824	Low
Sanogo et al., 2003	1	1	0	1	0	0	0	1	0	1	0	1	1	1	1	0	0	0.5294118	Moderate
Sarih et al., 2008	1	1	0	1	0	0	1	1	0	1	0	1	1	1	0	0	0	0.5294118	Moderate
Sarin et al., 2005	1	1	0	1	0	0	1	1	0	1	1	1	1	1	0	0	0	0.5882353	Moderate
Selmi et al., 2020 (a)	1	1	1	1	1	0	1	1	1	1	1	1	1	1	0	1	1	0.8823529	Low
Selmi et al., 2020 (b)	1	1	1	1	1	0	1	1	1	1	1	1	1	1	0	1	1	0.8823529	Low
Selmi et al., 2019 (a)	1	1	1	1	1	0	1	1	1	1	0	1	1	1	0	1	1	0.8235294	Low
Selmi et al., 2019 (b)	1	1	1	1	1	0	1	1	1	1	1	1	1	1	0	1	1	0.8823529	Low
Sfar et al., 2008	1	1	0	1	0	0	1	1	0	1	1	1	0	1	0	0	0	0.5294118	Moderate
Shuaib et al., 2020	1	1	0	1	0	0	1	1	0	1	0	1	1	1	1	1	0	0.6470588	Moderate
Socolovschi et al., 2012	1	1	0	1	0	0	1	1	0	1	1	1	1	1	0	0	0	0.5882353	Moderate
Socolovschi et al., 2007	0	0	0	1	0	0	1	1	0	1	1	1	1	1	1	0	0	0.5294118	Moderate
Sulyok et al., 2014	1	1	0	1	0	0	1	1	0	0	0	1	0	1	0	1	0	0.4705882	High
Teshale et al., 2015	1	1	0	1	0	0	1	1	0	0	0	1	0	1	0	0	0	0.4117647	High
Teshale et al., 2016	1	1	0	1	0	0	1	1	0	1	1	1	1	1	1	1	1	0.7647059	Low
Tomassone et al., 2016	0	0	0	1	0	0	1	1	1	1	0	1	1	1	1	0	0	0.5294118	Moderate
Tucker et al., 2021	0	0	0	1	0	0	0	1	0	1	0	1	1	1	1	1	0	0.4705882	High
Tufa et al., 2021	1	1	0	1	0	0	1	1	0	0	0	1	0	1	0	1	1	0.5294118	Moderate
Vanegas et al., 2018	0	0	0	1	0	0	0	1	1	1	1	1	1	1	1	1	0	0.5882353	Moderate
Wang’ang’a Oundo et al., 2020	1	1	0	1	0	0	1	1	0	1	1	1	1	1	0	1	1	0.7058824	Low
Yssouf et al., 2014	1	1	0	1	0	0	1	1	1	1	1	1	1	1	0	1	0	0.7058824	Low

Pub_authors	Q1	Q2	Q3	Q4	Q5	Q6	Q8	Q9	Q10	Q11	Q12	Q15	Q16	Q17	Q18	Q19	Q20	TOT	Risk bias
Znazen et al., 2013	1	1	0	1	0	0	1	1	0	1	1	1	1	1	1	1	1	0.7647059	Low
Iweriebor et al., 2017 (a)	1	1	0	1	0	0	1	1	0	1	0	0	1	1	1	1	1	0.6470588	Moderate
Iweriebor et al., 2017 (b)	0	0	0	1	0	0	0	1	0	1	1	0	1	1	1	1	1	0.5294118	Moderate
Abdullah et al., 2022	1	1	0	1	0	0	1	1	0	1	0	1	0	1	0	0	1	0.5294118	Moderate
AL-Hosary et al., 2021	1	1	0	1	0	0	1	1	0	0	0	0	0	0	0	1	0	0.3529412	High
Aouadi et al., 2022	1	1	0	1	0	1	1	1	0	1	1	1	1	1	0	1	1	0.7647059	Low
Benyahia et al., 2022	1	1	0	1	0	0	1	1	0	1	1	1	1	1	0	1	1	0.7058824	Low
Chitanga et al., 2021	1	2	0	0	0	0	1	1	1	1	0	1	0	1	0	1	0	0.5882353	Moderate
Elelu et al., 2022	0	0	0	0	0	0	0	1	0	1	0	1	0	1	0	1	1	0.3529412	High
Hegab et al., 2022	0	0	1	0	0	0	0	1	1	1	0	1	0	1	0	1	1	0.4705882	High
Hornok et al., 2022	1	1	0	1	0	0	1	1	0	1	0	1	0	1	0	0	0	0.4705882	High
Mediannikov et al., 2014	1	1	0	1	0	0	1	1	1	1	1	1	1	1	1	1	0	0.7647059	Low
Nimo-Paintsil et al., 2022	1	1	0	0	0	0	1	1	1	1	1	1	1	1	1	1	1	0.7647059	Low
Palomar et al., 2022	0	0	0	0	0	0	0	1	0	0	0	1	0	1	0	1	1	0.2941176	High
Qiu et al., 2021	1	1	0	1	0	0	1	1	0	1	1	1	1	1	1	1	0	0.7058824	Low
Said et al., 2021	1	1	0	1	0	0	1	1	1	1	1	1	1	1	0	1	1	0.7647059	Low

1 means “yes” while 0 means “no”.
